# Case Report: Revealing the truth: how superficial ossification mimics cutaneous tuberculosis in ossifying fibromyxoid tumor

**DOI:** 10.3389/fonc.2026.1880459

**Published:** 2026-08-28

**Authors:** Chao Wang, Hong Li

**Affiliations:** Department of Burn and Plastic Surgery, West China School of Medicine, Sichuan University, Sichuan University Affiliated Chengdu Second People’s Hospital, Chengdu Second People’s Hospital, Chengdu, China

**Keywords:** acid-fast bacilli, cutaneous tumor, false-positive, ossifying fibromyxoid tumor, tuberculosis

## Abstract

Ossifying Fibromyxoid Tumor (OFMT) is a rare mesenchymal neoplasm of intermediate biological potential that typically presents as a slow-growing mass. We report a diagnostic challenge involving a 54-year-old male with a 20-year history of a thigh mass that recently exhibited rapid enlargement and ulceration. Initial superficial biopsy led to a high suspicion of cutaneous tuberculosis due to false-positive acid-fast bacilli (AFB) staining and a positive T-SPOT, despite negative tuberculosis serology and normal inflammatory markers. This diagnostic pitfall was attributed to the “shell” of superficial ossification, which caused sampling error and nonspecific dye retention mimicking mycobacteria. Computed tomography (CT) was pivotal in resolving this discrepancy by revealing a characteristic “core–shell” pattern—a well-defined soft tissue tumor core beneath a dense ossified layer. Following a multimodal diagnostic integration, the patient underwent wide local excision with a 2.0 cm margin and reconstruction via split-thickness skin grafting. Histopathology and immunohistochemistry, showing partial loss of INI-1 expression, confirmed the diagnosis of OFMT. No recurrence or metastasis was observed during a 2-year follow-up. This case underscores the necessity of imaging to bypass biopsy limitations imposed by superficial ossification and highlights the risk of inappropriate anti-tuberculosis therapy when microbiological morphology conflicts with the broader clinical and radiological picture.

## Highlights

This case highlights how superficial ossification in an Ossifying Fibromyxoid Tumor can lead to biopsy sampling errors and false-positive acid-fast bacilli staining, mimicking cutaneous tuberculosis. By utilizing CT imaging to identify the underlying "core–shell" tumor structure, clinicians can avoid inappropriate anti-tuberculosis treatment and ensure timely radical surgical excision.

## Introduction

Ossifying Fibromyxoid Tumor (OFMT) is a rare soft tissue tumor characterized by fibromyxoid stroma and peripheral ossification (1, 2). It is classified as a neoplasm of intermediate biological potential due to its risk of local recurrence and occasional metastasis (3). Typically, OFMT presents as a slow-growing mass; however, atypical features, such as ulceration, necrosis, and calcification, may occur, particularly in long-standing lesions (4). These secondary changes can obscure the primary tumor architecture, complicating diagnosis, especially when only a superficial biopsy is performed. False-positive acid-fast bacilli (AFB) staining has been reported in non-infectious conditions, where dystrophic calcification within necrotic tissue may lead to nonspecific dye retention, mimicking mycobacterial organisms (5). Diagnostic pitfalls caused by the “shell” of superficial ossification in OFMT have rarely been described. We report a case of cutaneous OFMT that clinically and histologically mimicked tuberculosis, highlighting the role of superficial ossification in biopsy sampling error and false-positive AFB staining.

## Case presentation

**Figure 1:**
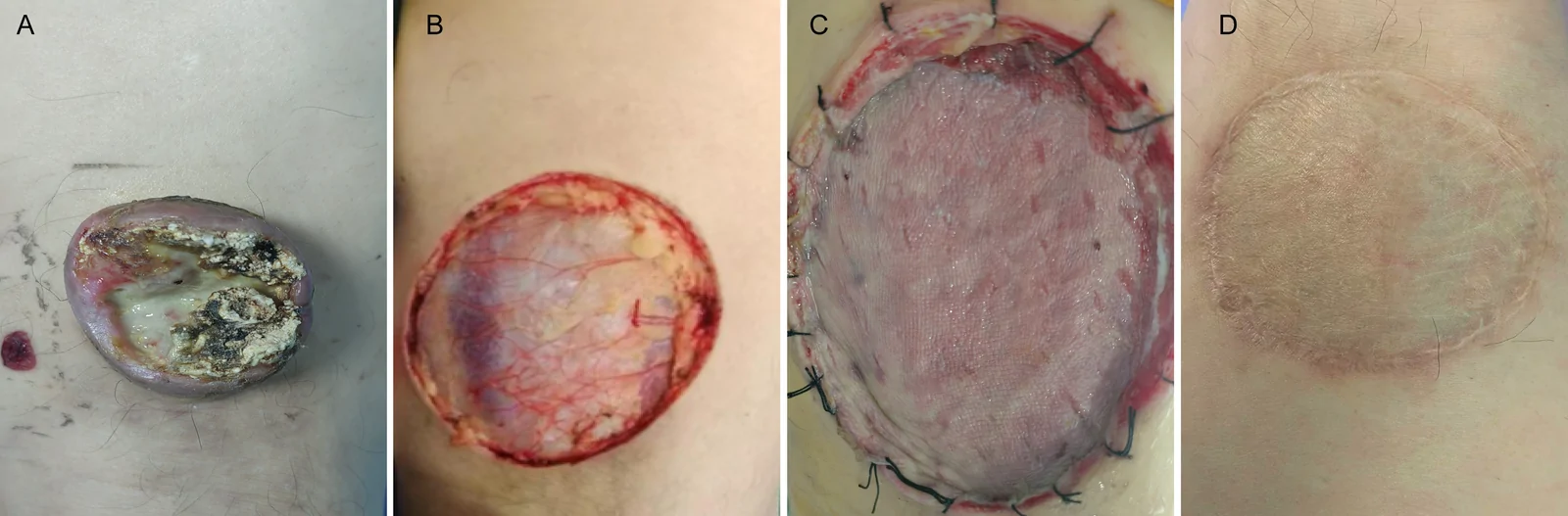
Clinical photographs of the lesion management and postoperative follow-up. **(A)** Preoperative view: An exophytic, ulcerated mass is seen on the right thigh. **(B)** Intraoperative view: Appearance after radical resection, showing a clean surgical bed. **(C)** 7-day postoperative view: The skin graft shows excellent initial survival with healthy wound margins. **(D)** 2-year postoperative view: Long-term follow-up demonstrates stable graft take, minimal scarring, and no signs of local recurrence.

A 54-year-old male presented with a 20-year history of a painless, slowly enlarging mass on the posterolateral aspect of his right thigh. Over the preceding year, the lesion exhibited rapid growth and developed surface ulceration. Physical examination revealed a 6.0 × 5.0 cm elevated plaque with a central ulcer, which felt stony-hard on palpation (**Figure 1A**). No regional lymphadenopathy was detected.

**Figure 2:**
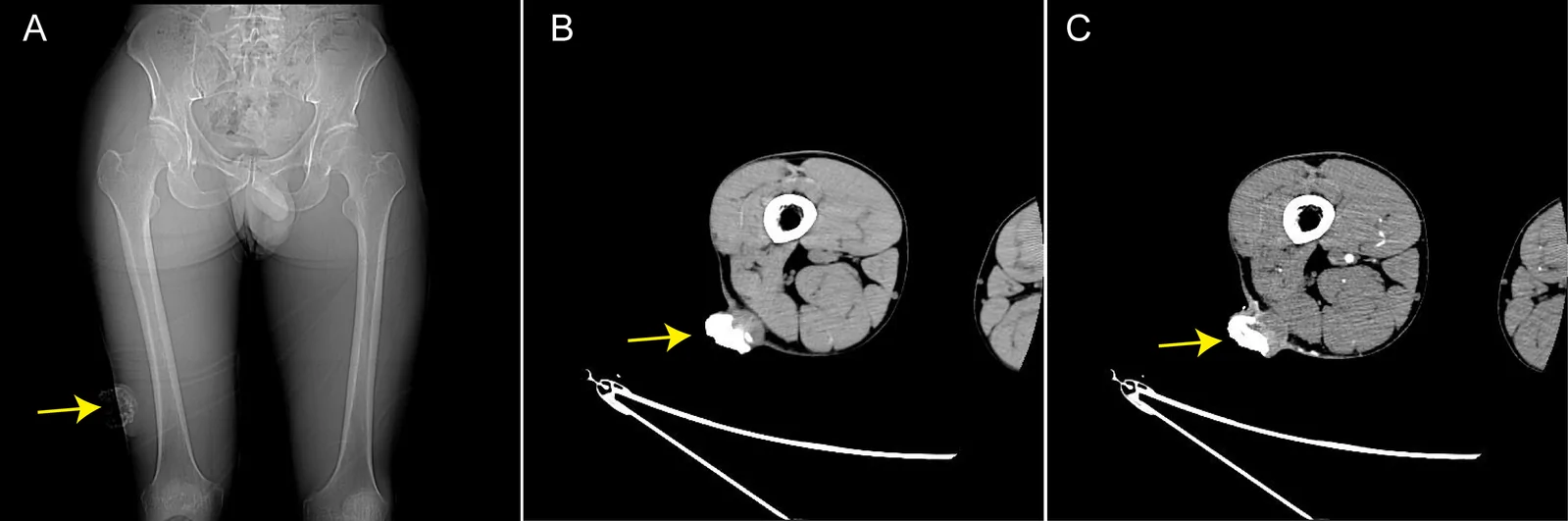
Radiographic and computed tomography (CT) evaluation of the lesion. **(A)** Scout view (topogram): A localized soft-tissue mass with internal stippled calcifications is observed on the lateral aspect of the right thigh (arrow). **(B)** Non-contrast CT: Axial slice showing a well-defined, exophytic lesion (arrow) with high-density calcified components. **(C)** Contrast-enhanced CT: Axial slice demonstrating the enhancement pattern of the lesion (arrow) and its relationship with the surrounding soft tissues.

Initial investigations pointed toward an infectious etiology in a tuberculosis-endemic region. The T-SPOT assay was positive. A preliminary superficial biopsy performed at our outpatient clinic revealed marked epidermal hyperplasia with extensive dermal calcium deposition and focal ossification. Ziehl–Neelsen (ZN) staining demonstrated focal dye retention within the calcified debris, which was reported as suspicious acid-fast bacilli (AFB), leading to a clinical suspicion of cutaneous tuberculosis. However, systemic evaluations, tuberculosis serology, and subsequent AFB smears from exudates were negative. However, tuberculosis serology remained negative, and subsequent AFB smears from the wound exudate were negative. While the C-reactive protein (CRP) level was within normal limits, and the systemic evaluation was otherwise unremarkable, the clinical picture remained localized and atypical for a chronic infection (6). Computed tomography (CT) provided crucial diagnostic information, revealing an exophytic lesion with central ulceration and a dense superficial ossification layer. Beneath this ossified layer, a well-circumscribed soft tissue mass measuring 4.5 × 2.9 cm was identified (**Figure 2**). This “core–shell” pattern suggested a neoplastic process rather than a primary infection.

Based on these findings, wide local excision was performed with a 2.0 cm lateral margin, extending to the superficial layer of the deep fascia (**Figure 1B**). The defect was reconstructed using a split-thickness skin graft (**Figure 1C**). Gross pathological examination of the excised specimen 5.5×4.0cm revealed a lobulated mass showing a firm, grey-white cut surface with areas of ossification. Histopathology confirmed short spindle-to-ovoid cells arranged in a lobulated pattern within a fibromyxoid stroma, flanked by prominent calcification and ossification (1, 7). All surgical margins—including 3-, 6-, 9-, and 12-o’clock lateral margins and the deep base margin—were completely free of tumor involvement (minimal deep margin distance: <0.1cm). Immunohistochemical analysis demonstrated strong positivity for S100 and CD10, partial positivity for CD99 and Desmin, and a low Ki-67 proliferation index (~3%). Crucially, nuclear expression of INI-1 (SMARCB1) showed partial loss. The tumor was negative for PCK, EMA, CD34, ERG, SMA, SATB2, SOX-10, NSE, and CEA. This immunophenotypic profile definitively established the diagnosis of Soft Tissue Ossifying Fibromyxoid Tumor (OFMT) (8). The postoperative course was uneventful, and the skin graft survived completely. No evidence of recurrence or metastasis was observed during the 2-year follow-up (**Figure 1D**).

## Discussion

This case illustrates a diagnostic pitfall where superficial ossification in OFMT led to both biopsy sampling error and false-positive AFB staining. In long-standing lesions, ossification may become dense and superficially located, forming a barrier that prevents biopsy instruments from reaching the tumor core. Consequently, only necrotic tissue or calcified debris above the ossified layer is sampled, leading to misinterpretation.

Although typical acid-fast bacilli demonstrate a characteristic beaded rod-like morphology, dystrophic calcifications and dense collagenous matrix in chronic ulcerative lesions can resistantly retain carbol fuchsin dye during acid-alcohol decolorization, generating microscopic artifacts. In this patient, the dense superficial ossified shell restricted the initial biopsy to the necrotic, calcified outer layer, preventing sampling of the underlying neoplastic core. Furthermore, the co-existing positive T-SPOT test likely represented latent tuberculosis infection (LTBI)—common in endemic regions—which, when combined with dye-retaining calcification artifacts, created a confounding diagnostic trap. Ultimately, imaging was pivotal in revealing the underlying tumor core, resolving the inconsistency between the initial superficial pathology and the actual clinical presentation.

The clinical imperative to distinguish OFMT from cutaneous tuberculosis is rooted in the diametrically opposed therapeutic paradigms. Cutaneous tuberculosis requires prolonged, systemic anti-tuberculosis chemotherapy, which carries risks of hepatotoxicity, multidrug resistance, and unnecessary medical burden. In contrast, OFMT is a surgical disease where medical therapy is ineffective. As demonstrated in this case, radical surgical intervention—specifically a wide local excision with clear margins—is the definitive treatment. Misinterpreting this neoplasm as a chronic infection not only leads to inappropriate medical management but also risks local tumor progression, potentially compromising future surgical salvage and increasing the complexity of reconstruction.

The partial loss of INI-1 expression observed in this case may be associated with atypical biological behavior and may explain the sudden progression of this previously indolent lesion (8). Complete surgical excision with adequate margins remains the treatment of choice (4). In this case, resection including the fascia followed by split-thickness skin grafting achieved favorable local control and functional recovery.

**Figure 3:**
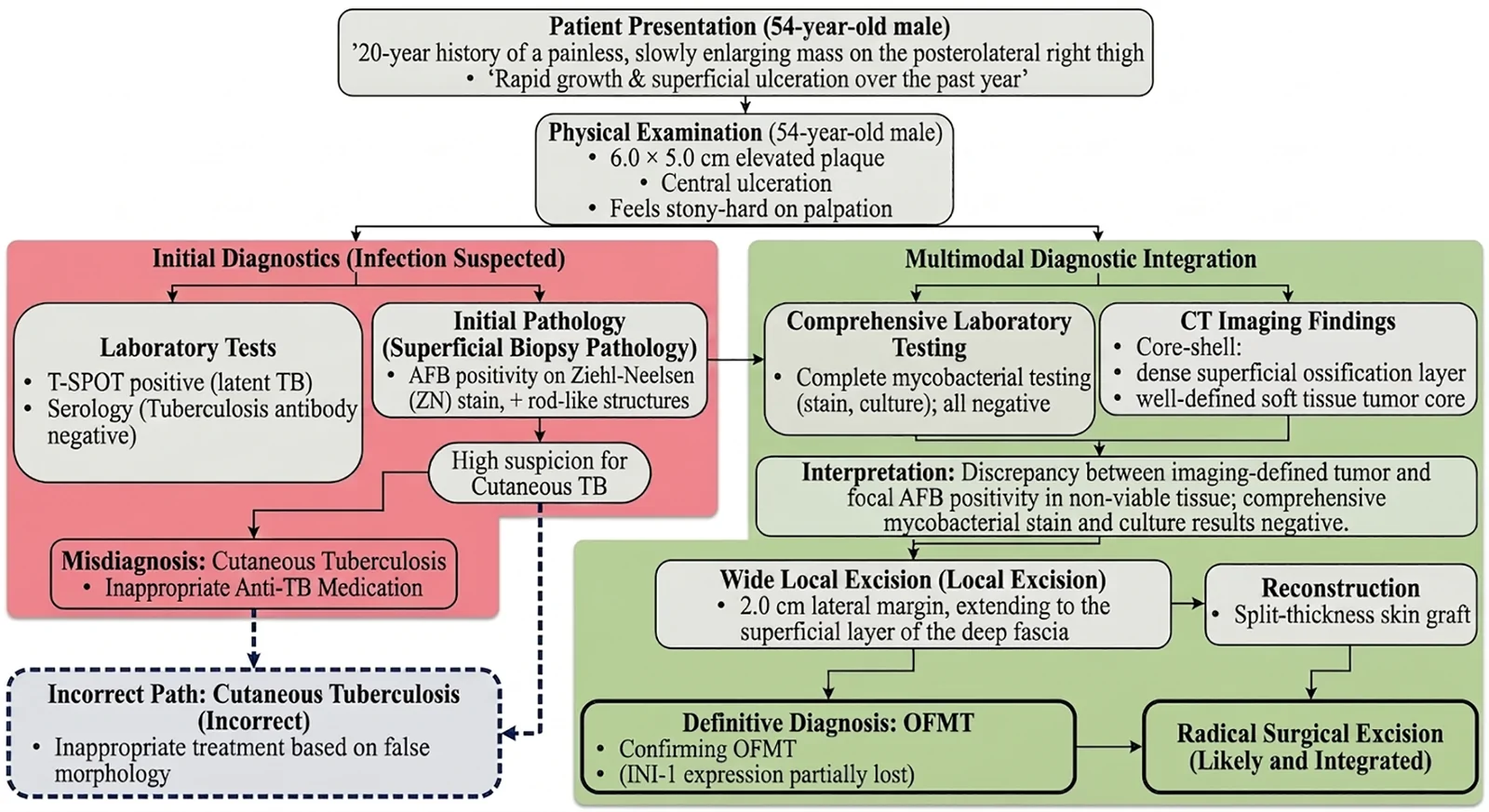
Clinical timeline of patient presentation, diagnostic evaluation, and surgical management. Summary of key milestones: initial presentation of a 20-year lesion with rapid 1-year ulceration, misleading outpatient workup (positive T-SPOT, initial ZN-positive superficial biopsy), definitive CT imaging revealing the ‘core–shell’ tumor architecture, radical local excision with split-thickness skin graft reconstruction, histopathological/IHC confirmation, and 2-year recurrence-free outcome.

In conclusion, superficial ossification in OFMT can lead to a cascade of diagnostic pitfalls, primarily biopsy sampling error and false-positive AFB staining. As illustrated in our diagnostic workflow (**Figure 3**), clinicians must maintain a high index of suspicion when microbiological findings conflict with immunological evaluations and the overall clinical picture. Early identification via CT imaging is essential to avoid ineffective anti-tuberculosis medication and ensure timely radical surgical excision, which remains the cornerstone of management for this intermediate-potential neoplasm.

## Data Availability

The original contributions presented in the study are included in the article/supplementary material. Further inquiries can be directed to the corresponding author.
